# NGSMHC: a simple bioinformatics tool for comprehensively typing major histocompatibility complex genes in non-human species using next-generation sequencing data

**DOI:** 10.5713/ab.25.0468

**Published:** 2025-09-30

**Authors:** Mingue Kang, Byeongyong Ahn, Jae Yeol Shin, Jongan Lee, Eun Seok Cho, Chankyu Park

**Affiliations:** 1 Department of Stem Cell and Regenerative Biotechnology, Konkuk University, Seoul, Korea; 2 Animal Genome & Bioinformatics, National Institute of Animal Science, Rural Development Administration, Wanju, Korea; 3 Swine Division, National Institute of Animal Science, Rural Development Administration, Cheonan, Korea

**Keywords:** Allele Typing, Major Histocompatibility Complex, Next-generation Sequencing

## Abstract

**Objective:**

Understanding the individual- and population-level polymorphisms of major histocompatibility complex (MHC) genes is crucial for identifying associations between MHC variations and immune phenotypes. To support this, we developed NGSMHC, a streamlined bioinformatics tool for efficient and accurate MHC genotyping using next-generation sequencing (NGS) data in non-human species.

**Methods:**

NGSMHC constructs phased haplotype contigs of selected MHC genes from BAM-format mapping data and determines the best matching MHC alleles and genotypes via nucleotide BLAST analysis against a user-provided reference set of MHC alleles. We evaluated NGSMHC using short-read whole-genome sequencing (WGS) data from 12 pigs, focusing on swine leukocyte antigen (SLA) genes. The typing results from NGSMHC were compared to those obtained using polymerase chain reaction sequence-based typing (PCR-SBT). In addition, we tested NGSMHC on a publicly available long-read WGS dataset with known SLA genotypes.

**Results:**

The short-read WGS data showed an average read depth of 20.9× across the SLA region, enabling typing of *SLA-2*, *SLA-3*, *SLA-DRB1*, and *SLA-DQB1* using NGSMHC. The concordance rates between NGSMHC and PCR-SBT were 88% for *SLA-3*, 92% for *SLA-DRB1*, and 100% for *SLA-DQB1*. However, *SLA-2* typing showed lower concordance (58%), likely due to its high sequence similarity with other SLA class I genes and complex intra-locus polymorphisms. In contrast, NGSMHC accurately identified all tested SLA genotypes—including *SLA-1*, *SLA-2*, *SLA-3*, *SLA-DRA*, *SLA-DRB1*, *SLA-DQA*, and *SLA-DQB1*—when applied to the long-read WGS data.

**Conclusion:**

NGSMHC is a simple and effective tool for MHC genotyping using NGS data, particularly for non-human species. Its accuracy is significantly improved by long-read sequencing, underscoring the importance of read length in precise MHC allele determination.

## INTRODUCTION

Major histocompatibility complex (MHC) is a cell surface membrane protein involved in immune activation in jawed vertebrates [1]. MHC molecules are primarily classified into class I and class II based on their structural characteristics and antigen presentation mechanisms [2]. Their main function is to present pathogen-derived peptide fragments on the cell surface, enabling T cells to recognize infected or abnormal cells and trigger immune responses [2]. Analyzing the genetic diversity of this region provides valuable insights into individual- and population-level disease resistance and susceptibility, as well as natural selection and evolutionary processes [3,4]. Furthermore, allelic diversity of MHC genes plays a crucial clinical role as a key determinant of tissue transplantation compatibility [5].

Despite the importance of assessing MHC gene diversity, determining the genetic variation of MHC genes remains challenging because of extensive polymorphisms and high sequence similarity among MHC family genes [6]. Several typing strategies utilizing cDNA and genomic DNA have been developed to identify sequence variations in MHC genes [6–8]. One of the most reliable methods for characterizing MHC alleles is polymerase chain reaction sequence-based typing (PCR-SBT), which involves amplifying target loci using specific primers, followed by sequencing to determine the allelic sequences [6,9]. However, despite its reliability, PCR-SBT has several limitations, including restricted sequencing coverage, separate typing for each gene, labor-intensive and expensive nature of its multistep experimental process [6,10]. The increasing availability of high-throughput whole-genome sequencing (WGS) data from diverse species necessitates the development of bioinformatic analysis tools to type MHC genes from available WGS datasets.

Various tools have been developed to simultaneously infer the MHC allele haplotypes of multiple MHC genes from next-generation sequencing (NGS) data, though their application has primarily focused on human MHC, known as human leukocyte antigen (HLA) [11–15]. Consequently, applying these tools to analyze MHC alleles in non-human species remains challenging because of their limited versatility regarding the use of reference allele databases for species other than humans and their requirement for high computing power [16].

In this study, we developed an NGS-based MHC typing tool, NGSMHC, that enables MHC typing of pigs and other non-human species using a user-generated reference allele FASTA file. When we applied this tool to our test dataset, using short-read WGS data from pigs, we obtained reliable typing results for 3 swine leukocyte antigen (SLA) genes: *SLA-3, SLA-DRB1*, and *SLA-DQB1*. In contrast, when typing was performed using the long-read WGS data, NGSMHC demonstrated accurate typing across all tested SLA genes (*SLA-1, SLA-2, SLA-3, SLA-DRA, SLA-DRB1, SLA-DQA*, and *SLA-DQB1*). Herein, we discuss the considerations and requirements for improving the accuracy of SLA typing using NGS data. Our work contributes to advancing the NGS-based MHC typing of species with limited data resources.

## MATERIALS AND METHODS

### Animals and DNA preparation

Twelve Woori Black pigs from a closed breeding system with a small population size (Duroc×Korean native pig crossbred) at the National Institute of Animal Science, RDA, Korea [17,18] were selected for this study. Genomic DNA was prepared from 3 mL of whole blood collected using the QIAamp DNA Blood Midi Kit according to the manufacturer’s protocol (QIAGEN).

### Whole-genome sequencing and read mapping

Genomic DNA (2 μg) was cleaved using a Covaris S2 instrument (Covaris) and a paired-end WGS library was prepared using a DNA PCR-Free Library Prep Kit (Illumina), according to the manufacturer’s protocol. WGS was performed using the NovaSeq 6000 platform (Illumina). The FASTQ sequencing reads were aligned to the current pig reference genome (Sscrofa11.1; RefSeq accession no. GCF_000003025.6) using BWA-MEM v0.7.18-r1243 [19]. For targeted refinement, reads mapped to the SLA cluster region on chromosomes 7:22,324,137–25,682,665 were extracted and realigned using the hash-based aligner Stampy v1.0.32 [20] with the options --bamkeepgoodreads --gapopen = 60 --apextend = 15. For long-read data, mapping was performed using minimap2 v2.28, with the -ax map-pb option [21].

### Preparation of major histocompatibility complex reference allele data

SLA allele references of all reported SLA genes in IPD, including *SLA-1*, *SLA-2*, *SLA-3*, *SLA-6*, *SLA-DRA*, *SLA-DRB1*, *SLA-DQA*, *SLA-DQB1*, and *SLA-DMA* were downloaded from the SLA data stored in the IPD-MHC database (www.ebi.ac.uk/ipd/mhc/group/SLA/) and a reference database file in the FASTA format was constructed. The reference allele sequences corresponded to exons 2 and 3 of SLA class I genes and exon 2 of SLA class II genes. For alleles with incomplete exon sequences, the missing regions were supplemented with the exon sequence of the allele with the highest sequence similarity. The numbers of alleles used in the reference database were 85, 94, 39, 5, 6, 91, 23, 45, and 2 for *SLA-1, SLA-2*, *SLA-3*, *SLA-6, SLA-DRA, SLA-DRB1*, *SLA-DQA, SLA-DQB1*, and *SLA-DMA*, respectively (Supplement 1).

### Experimental typing of swine leukocyte antigen genes

Typing and allele differentiation for *SLA-2*, *-3*, *SLA-DQB1*, and *SLA-DRB1* were conducted as previously described [22–24]. Briefly, specific amplifications of both exons 2 and 3 for SLA class I and exon 2 for SLA class II gene were achieved using target specific primer sets using 25 ng of genomic DNA, 1 μM of locus-specific primers, 250 μM dNTPs, 1 unit of Supertherm Taq DNA polymerase (JMR Holdings), and 10× reaction buffer containing 15 mM MgCl_2_ using an ABI9700 thermocycler (Applied Biosystems). The reactions were conducted under an initial denaturation step at 94°C for 3 min; 30 cycles of denaturation at 94°C for 30 s, annealing at the locus-specific primer annealing temperature for 45 s, and extension at 72°C for 90 s; followed by a final extension step at 72°C for 10 min. 5 μL of the PCR product was subjected to direct sequencing using the BigDye Terminator v3.1 Cycle Sequencing Kit (Applied Biosystems) with 2 pmol of a locus-specific sequencing primer under the following conditions: pre-denaturation at 96°C for 1 min, followed by 25 cycles of 96°C for 10 s, 50°C for 5 s, and 60°C for 4 min. The sequencing reaction products were purified using ethanol precipitation, resuspended in 10 microliters of Hi-Di Formamide (Applied Biosystems), and analyzed on an ABI3730 DNA Analyzer (Applied Biosystems). All primer sequences used in the PCR-SBT are listed in Supplement 2.

### Bioinformatic tools and data

The bioinformatic tools used in this study were as follows: SAMtools v1.14 [19] for BAM handling and indexing; bcftools v1.6 [25] and longshot v1.0 [26] for variant calling of short- and long-read sequencing data, respectively; whatshap v2.3 [27] for haplotype phasing of variant calling data, bedtools v2.29.1 [28] for genome sequence extraction; and NCBI BLASTN v2.15.0 [29] for nucleotide blast of the generated contigs to the MHC references. All the code scripts were written in python v3.8 (www.python.org) and deposited in the National Center for Biotechnology Information (NCBI; www.ncbi.nlm.nih.gov) database. The Sequence Read Archive (SRA; www.ncbi.nlm.nih.gov/sra) accession number for the long-read sequencing data is SRR25949611.

## RESULTS

### Generation of whole genome sequencing data for 12 pigs

We generated short-read WGS data from 12 Woori Black pigs. On average, each sample generated 55,637,663,900 bases from 368,461,350 reads (Supplement 3). Across the 12 FASTQ files, 90.34% of the bases had Phred quality scores above 30, with a mean Phred score of 35.39. The sequences were submitted to the SRA database under the following biosample accession numbers: SAMN48396345, SAMN48396346, SAMN48396347, SAMN48396356, SAMN48396351, SAMN48396348, SAMN48396349, SAMN48396350, SAMN48396352, SAMN48396353, SAMN48396354, and SAMN48396355.

### Overall pipeline of the development of a new next-generation sequencing-based swine leukocyte antigen typing tool using NGSMHC

We developed a WGS-based MHC typing tool, NGSMHC, with the characteristics of versatility in using reference allele data and low computing power requirements. The tool consists of three Python scripts. First, *MHCReadFilter.py* performs a BLASTN of mapped reads of the input BAM against the SLA allele reference and filters out misaligned reads, generating a filtered BAM file (output.filt.bam file in the output_bam directory). Next, *NGSMHC.py* uses this filtered BAM, along with the pig reference genome, to perform variant calling and haplotype phasing to produce a phased VCF file. Finally, *MHCContigGenotyper.py* generates haplotype contigs containing SLA allele information based on the phased VCF and conducts BLASTN against the target SLA allele reference to call the final SLA genotype. *NGSMHC.py* serves as the main script that imports and runs functions from both *MHCReadFilter.py* and *MHCContigGenotyper.py*. SLA typing from NGS data can be easily performed using a single command through *NGSMHC.py* with the following commands: ./NGSMHC.py -b [input BAM] -m [location of the SLA reference directory] -t [target SLA name] -g [reference genome]. The underlying bioinformatics processes of the NGSMHC are described below.

### BAM filtering

**Figure 1:**
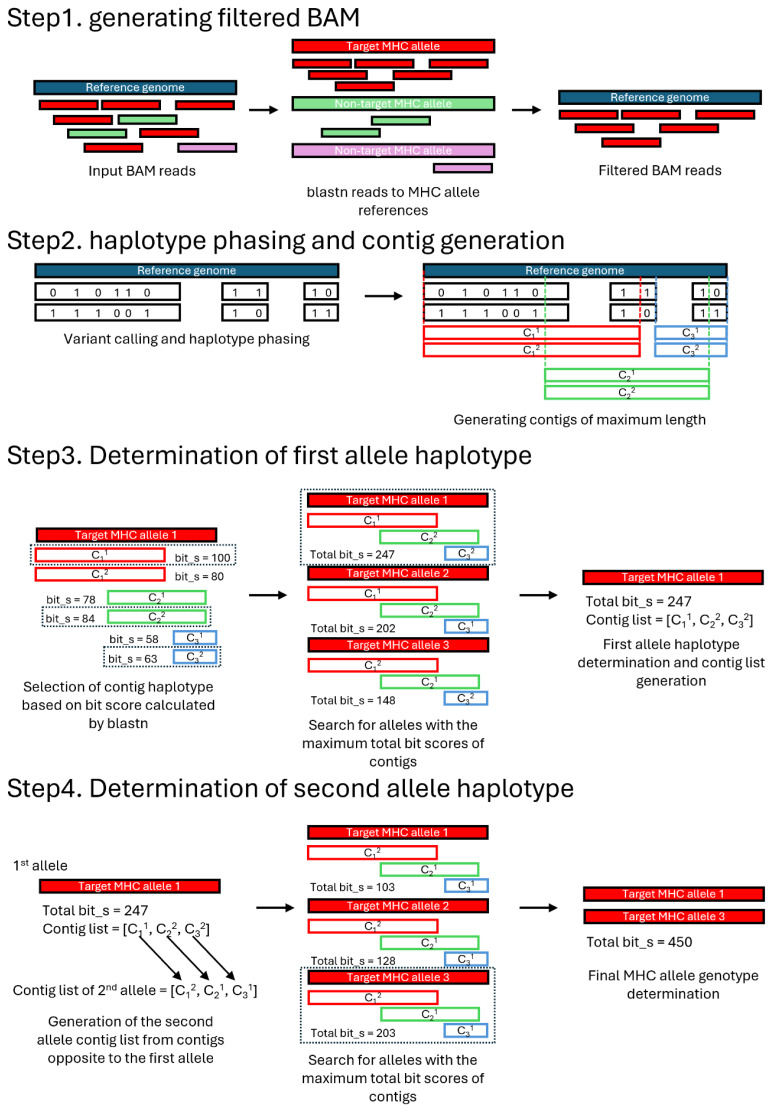
Overall pipeline of the NGSMHC workflow. A schematic overview of the NGSMHC workflow for MHC allele typing using input BAM files. Detailed descriptions of each step are provided in the Results section. NGS, next-generation sequencing; MHC, major histocompatibility complex.

To further remove non-specifically mapped reads from the target gene, despite originating from paralogs of the target gene owing to high sequence similarities, BLAST analysis was conducted iteratively using the mapped reads as queries against reference allele sets of paralogous SLA genes as subjects. For instance, reads mapped to *SLA-3* were subjected to BLAST analysis against reference alleles of other SLA class I genes, such as *SLA-1* and *SLA-2*. In Step 1 of Figure 1, BLAST analysis is performed to evaluate the alignment of target reads. Reads that exhibit a higher bit score when aligned to a non-target gene’s reference allele—compared to the target gene—are considered off-target and subsequently removed from the BAM file. The resulting filtered BAM file is then saved in the output_bam directory under the filename output.filt.bam. This process is performed using the script file *MHCReadFilter.py*. For datasets in which additional read filtering is unnecessary, such as long-read or targeted sequencing, this filtering process can be disabled using the--disable_read_filtering option in *NGSMHC.py*.

### Variant calling and haplotype phasing

To generate the output.filt.bam file, NGSMHC is used to perform variant calling on the BAM file using the bcftools mpileup for short- and long-read sequencing data. The command lines are “bcftools mpileup -BAOu -f [reference_genome] -r [target_region] output_bam/output. filt.bam | bcftools call -mv -Oz -o output_vcf/tmp. output.vcf.gz” and “longshot --ref [reference_genome] -r [target_region] -F --bam output_bam/output. filt.bam --out output_vcf/tmp. output.vcf” to execute the variant calling process for short- and long-read data, respectively. Subsequently, the tmp.output.vcf file generated by the longshot variant caller is compressed into tmp.output.vcf.gz using bgzip. The resulting VCF file, tmp.output.vcf.gz, is subjected to haplotype phasing using WhatsHap v2.3. The polyphase-p 2 option is applied for haplotype phasing. The phased VCF file is saved as an output.vcf file in the output_vcf directory (Step 2 of Figure 1).

### Generation of haplotype contigs and allele determination

*MHCContigGenotyper.py* groups called variants within the same haplotype block based on the PS tag designated by WhatsHap. Additionally, variant calls are clustered from the phased VCF file and the longest possible haplotype contigs for each haplotype block is constructed. The resulting haplotype contigs are provided in the “output.contig.fa” file. The two haplotype-phased contigs within a haplotype block are designated haplotype contig 1 (C_n_^1^, where *n* represents the block number) and haplotype contig 2 (C_n_^2^) in the heterozygous state. For example, the two haplotypes of the first contig within the target locus are designated C_1_^1^ and C_1_^2^ (Step 2 of Figure 1). Subsequently, the phased contigs are sequentially BLASTed against each reference allele of the target MHC gene for all haplotype blocks spanning the target MHC gene. The name of the contig with the highest bit score against a given reference allele in the BLAST analysis is stored in the Contig List, along with the corresponding reference allele name, bit score, and percent identity by BLASTN. This process is repeated for all phased contigs within the haplotype blocks that constituted the target MHC gene.

After determining the reference allele that best matched a given contig within each haplotype block, the total bit score for all possible combinations of contig assemblies of the target MHC genes is calculated. This is performed by summing the bit scores of each contig from the haplotype blocks listed on the Contig List. The allele with the highest total bit score is designated as “Allele 1” (Step 3 of Figure 1). To identify the second allele (“Allele 2”), a new Contig List is generated using the remaining haplotype contigs that undergoes the same identification process as Allele 1 (Step 4 of Figure 1). The final typing result is presented as a pair of alleles consisting of Allele 1 and 2, along with their total bit scores and average percent identity to the matching reference alleles (Supplement 4).

### Successful typing of *SLA-3*, *SLA-DQB1*, and *SLA-DRB1* using NGSMHC

Typing of four major SLA genes (*SLA-2*, *SLA-3*, *SLA-DRB1*, and *SLA-DQB1*) was performed using the NGSMHC and WGS of 12 pigs. The results showed the presence of eight, four, four, and three alleles for *SLA-2*, *SLA-3*, *SLA-DRB1*, and *SLA-DQB1*, respectively. The small number of alleles observed in the SLA typing coincided with the genetic nature of the animal population. For two animals (W4217 and W4550), SLA-2*04:05 was inferred together with *04:02*, with differences in positions 12 (C→A) and 168 (T→A), with exon 3 between them (Table 1), indicating that similar alleles can be inferred together in analysis conducted using NGSMHC.

In addition, PCR-SBT was conducted for the four SLA genes and the results were compared with those of NGS-based typing (Table 1). Complete concordance (24 concordant calls out of 24 total allele calls) was observed for *SLA-DQB1*, with high rates for *SLA-DRB1* (96%, 23/24) and *SLA-3* (86%, 21/24). However, the concordance was only 58% (14/24) for *SLA-2*. As SLA typing results obtained using PCR-SBT proved to be comprehensive and highly accurate [22–24], we believe that the discrepancies in the typing results between NGSMHC and PCR-SBT are likely due to typing errors in WGS-based SLA typing. This indicates that the accuracy of WGS-based typing of SLA genes is heavily influenced by the complexity of the loci subjected to typing, which is attributed to the extent of polymorphism and sequence homology to paralogs in the region.

### Low typing accuracy for *SLA-2* due to misalignment of swine leukocyte antigen class I genes

The nucleotide sequence differences of exons 2 and 3 among *SLA-1*, *SLA-2*, and *SLA-3* were reported to be only 7.82%–8.79% for the SLA system [30]. Therefore, high sequence homology among paralogous SLA classical class I genes can result in read misalignments with the target. For example, index values for read-mapping quality such as the number of reads and per-base read depth were significantly lower for *SLA-2* exon 2 (averages of 12.8 and 5.4) compared with those of other SLA regions (45.4–61.3 and 16.1–24.5, respectively). In contrast, the values of *SLA-2* exon 3 were much higher (average of 108.3 and 42.0) than those of other regions (Supplement 5). Additionally, the breadth of coverage, which represents the percentage of the target region covered by sequencing reads, only failed to reach 100% in *SLA-2* exon 2, with an average coverage of 97.3% (Supplement 5). The rest of the parameters, including mean sequencing quality score (Q = 34.8–35.7) and mapping quality (MAPQ = 38.3–66.7), were not significantly different among the loci (Supplement 5). The results of the BLAST analysis using 94 *SLA-2* reference alleles from the pig genome (Sscrofa11.1) showed that only 56% (53/94) of *SLA-2* exon 3 and 23% (22/94) of *SLA-2* exon 2 sequences showed the highest bit score in the corresponding region (Supplement 6), consistent with the low typing accuracy of *SLA-2*. For example, for W5161, only three reads were properly mapped to the *SLA-2* region among the 27 reads, with >99% sequence identity to the actual allele SLA-2*16:03, whereas the rest were mapped to *SLA-1*, *SLA-3*, or *SLA-7* (Supplement 7). This suggests that the high similarity among SLA class I-related sequences in the genome is an inherent challenge for short-read NGS-based SLA typing.

### Improvement of typing accuracy using long-read sequencing data

The typing accuracy for *SLA-2* of NGSMHC was less reliable than expected for short-read WGS data, we conducted SLA typing for seven SLA genes, *SLA-1, SLA-2*, *SLA-3, SLA-DRA*, *SLA-DRB1, SLA-DQA*, and *SLA-DQB1*, using publicly available PacBio CLR long-read WGS data (SRA accession: SRR25949611) to evaluate the effect of read length on typing accuracy. The typing results obtained were homozygous for SLA-1*14:02, SLA-2*11:02, SLA-3*04:03, SLA-DRA*02:02:03, SLA-DRB1*05:01, SLA-DQA*01:03, and SLA-DQB1*08:01, which showed complete concordance with the reported SLA types of all loci, except *SLA-2* [31]. However, further analysis of the reads mapped to the target region suggested a possible error in the reported typing result of *SLA-2*, suggesting that our typing result (SLA-2*11:04 or 11:05 homozygote) was correct (Table 2, Supplement 8). SLA-2*11:04 and *11:05* had identical sequences in exons 2 and 3, respectively. These results indicate that the locus complexity of SLA class I genes can be overcome using long-read sequences as input data. Typing of *SLA-1* was possible because the typed individuals did not carry *SLA-1* duplications. The read mapping result of target exon regions of the seven SLA genes to the SLA region of the pig reference genome exhibited a read distribution of 12 (*SLA-DRB1* exon 2) to 23 (*SLA-3* exon 2) in terms of the number of reads for long-read sequences. The breadth of coverage for all target regions was 100% and the average read depth per base was 10.8 (*SLA-DRB1* exon 2) to 21.9 (*SLA-3* exon 2). The average read MAPQ ranged from 58.0 (*SLA-1* exon 2) to 60.0 (*SLA-2* exons 2 and 3, *SLA-DRA* exon 2, *SLA-DRB1* exon 2, *SLA-DQA* exon 2) (Supplement 9). Therefore, our results indicated that SLA typing using NGSMHC with long-read WGS can substantially enhance typing accuracy.

## DISCUSSION

Several NGS-based MHC typing tools that use different strategies are available. Some map sequencing reads directly to MHC allele references to prevent errors in the genome mapping step and improve typing accuracy (e.g., Optitype [11], Polysolver [13], xHLA [32], HLA-HD [14]). Others, such as HLA*LA and MHC-PRG, construct a population reference graph (PRG) based on the sequence information of multiple MHC alleles and haplotype data to determine MHC genotypes [12,15]. These tools have over 90% typing accuracy for *HLA-A*, *HLA-B*, *HLA-C*, *HLA-DRB1*, and *HLA-DQB1* genes [16]. However, all showed limitations in their applications, including restricted applications to specific classes of MHC genes (e.g., Optitype and Polysolver), limited applicability only to HLA, and demanding high-end computational power (e.g., Optitype, HLA*LA, and HLA-HD). In non-human species, AmpliSAS-based typing of MHC genes using NGS analysis of target-specific amplicons has demonstrated considerable typing accuracy [33–36]. However, this approach requires target-specific amplicons to reduce the complexity of MHC gene sequencing, rather than the simultaneous typing of NGSMHC.

The results of SLA typing for our test dataset showed a lower typing accuracy for *SLA-2* because of the high sequence homology among SLA class I paralogs. Locus specific regions for classical SLA class I genes were identified at the 5′ UTR region of SLA class I genes and used for specific amplification of SLA genes using PCR [23]. Our results showed that the generation of locus-specific contigs using WGS short-reads can be achieved for SLA class II genes, but it is extremely challenging to perform this for SLA class I genes. Despite the high cost of long-read sequencing, the use of long-read sequences (>3kb) could solve the problems associated with short-read NGS-based typing, including *SLA-2*, as demonstrated in our results. Furthermore, this has been demonstrated in various MHC typing studies that used long-read sequencing data from various species [37,38].

To achieve the desired accuracy in MHC typing from short-read NGS, an appropriate read length, sequencing depth, and optimization of the data through additional processing can improve the mapping accuracy. A recent study on the 24 MHC genes of koalas using short-read WGS data with a 150 bp read length determined that a minimum sequencing depth of 20×, with an optimal depth of ≥ 30×, is required for reliable variant calling [39].

Consistently, samples W4217 and W5721, which has a lower average depth than the others in our dataset, showed typing discrepancies even for *SLA-3* and *SLA-DRB1*, indicating the importance of the depth of mapped reads in this region (Table 1, Supplement 5). Additionally, the methods used for read mapping and variant calling may have affected the results [40]. Furthermore, the contig generation function of NGSMHC is useful for identifying novel alleles that are absent from reference alleles.

This study measured the SLA typing performance of NGSMHC using a small population of 12 Woori Black pigs, a single breed of Duroc × Korean native pig crossbreds. Accordingly, the diversity of SLA alleles identified in this Woori Black pig population was found to be quite limited, representing only a small fraction of the actual SLA allele diversity in the pig population (Table 1). Therefore, further analysis will be required in the future to expand the study to include more breeds and confirm whether appropriate typing results are obtained for alleles not identified in our results.

## CONCLUSION

In this study, we developed NGSMHC, an NGS-based MHC typing tool suitable for multiple species. This tool performs MHC typing through steps consisting of filtering misaligned reads from the input BAM file, variant calling, haplotype phasing, haplotype contig generation, and final allele determination by blasting the generated contigs with MHC allele references. Implementation of NGSMHC to analyze short-read WGS data from pigs showed successful typing of *SLA-3, SLA-DRB1*, and *SLA-DQB1*. NGSMHC analysis of long-read WGS data demonstrated sufficient typing accuracy for all major SLA genes compared with short-read data. Analysis of the mistyped results indicated that typing performance was significantly influenced by the accuracy of read mapping and variant calling. This tool could be utilized for NGS-based MHC typing in species with no proper typing tool for MHC genes, and it has advantages such as simplicity and species versatility, as well as the easy preparation of reference alleles.

## Figures and Tables

**Table 1:** Results of SLA typing of 12 Woori-black pigs

Sample	Biosample acc.	Method	*SLA-2*		*SLA-3*		*SLA-DRB1*		*SLA-DQB1*	

			Allele 1	Allele 2	Allele 1	Allele 2	Allele 1	Allele 2	Allele 1	Allele 2
W4052	SAMN48396345	PCR-SBT	04:01	04:02:01 or 04:02:02	04:01:02 or 04:01 or 04:06	04:01:02 or 04:01 or 04:06	02:01:01	05:01 or 05:03	02:01 or 02:05	02:01 or 02:05
		NGSMHC	04:01	04:02:01 or 04:02:02	04:01:02 or 04:01 or 04:06	04:01:02 or 04:01 or 04:06	02:01:01	05:01 or 05:03	02:01 or 02:05	02:01 or 02:05
W4217	SAMN48396346	PCR-SBT	16:03	04:02:01 or 04:02:02	04:01:02 or 04:01 or 04:06	05:03:01 or 05:03:02 or 05:04	01:01	05:01 or 05:03	01:01	02:01 or 02:05
		NGSMHC	01:02	04:02:01 or 04:02:02 or 04:05	04:01:02 or 04:01 or 04:06	04:01:02 or 04:01 or 04:06	01:01	01:01	01:01	02:01 or 02:05
W4223	SAMN48396347	PCR-SBT	16:03	04:02:01 or 04:02:02	04:01:02 or 04:01 or 04:06	05:03:01 or 05:03:02 or 05:04	01:01	05:01 or 05:03	01:01	02:01 or 02:05
		NGSMHC	01:02	07:04	04:01:02 or 04:01 or 04:06	05:03:01 or 05:03:02 or 05:04	01:01	05:01 or 05:03	01:01	02:01 or 02:05
W5161	SAMN48396356	PCR-SBT	04:01	16:03	04:01:02 or 04:01 or 04:06	05:03:01 or 05:03:02 or 05:04	01:01	02:01:01	01:01	02:01 or 02:05
		NGSMHC	04:01	04:09	04:01:02 or 04:01 or 04:06	05:03:01 or 05:03:02 or 05:04	01:01	02:01:01	01:01	02:01 or 02:05
W5162	SAMN48396351	PCR-SBT	04:01	16:03	04:01:02 or 04:01 or 04:06	05:03:01 or 05:03:02 or 05:04	01:01	02:01:01	01:01	02:01 or 02:05
		NGSMHC	04:01	04:05	04:01:02 or 04:01 or 04:06	05:03:01 or 05:03:02 or 05:04	01:01	02:01:01	01:01	02:01 or 02:05
W4235	SAMN48396348	PCR-SBT	04:02:01 or 04:02:02	04:03	04:01:02 or 04:01 or 04:06	05:03:01 or 05:03:02 or 05:04	02:01:01	05:01 or 05:03	02:01 or 02:05	02:01 or 02:05
		NGSMHC	04:02:01 or 04:02:02	04:01	04:01:02 or 04:01 or 04:06	05:03:01 or 05:03:02 or 05:04	02:01:01	05:01 or 05:03	02:01 or 02:05	02:01 or 02:05
W4550	SAMN48396349	PCR-SBT	04:02:01 or 04:02:02	15:01	04:01:02 or 04:01 or 04:06	03:03	02:01:01	11:01	02:01 or 02:05	05:03
		NGSMHC	04:02:01 or 04:02:02	04:02:01 or 04:02:02 or 04:05	04:01:02 or 04:01 or 04:06	03:03	02:01:01	11:01	02:01 or 02:05	05:03
W4937	SAMN48396350	PCR-SBT	04:02:01 or 04:02:02	04:02:01 or 04:02:02	04:01:02 or 04:01 or 04:06	04:01:02 or 04:01 or 04:06	02:01:01	02:01:01	02:01 or 02:05	02:01 or 02:05
		NGSMHC	04:02:01 or 04:02:02	04:02:01 or 04:02:02	04:01:02 or 04:01 or 04:06	04:01:02 or 04:01 or 04:06	02:01:01	02:01:01	02:01 or 02:05	02:01 or 02:05
W5226	SAMN48396352	PCR-SBT	04:02:01 or 04:02:02	16:03	04:01:02 or 04:01 or 04:06	05:03:01 or 05:03:02 or 05:04	01:01	02:01:01	01:01	02:01 or 02:05
		NGSMHC	04:09	16:03	04:01:02 or 04:01 or 04:06	05:03:01 or 05:03:02 or 05:04	01:01	02:01:01	01:01	02:01 or 02:05
W5621	SAMN48396353	PCR-SBT	04:02:01 or 04:02:02	04:02:01 or 04:02:02	04:01:02 or 04:01 or 04:06	04:01:02 or 04:01 or 04:06	02:01:01	02:01:01	02:01 or 02:05	02:01 or 02:05
		NGSMHC	04:02:01 or 04:02:02	04:02:01 or 04:02:02	04:01:02 or 04:01 or 04:06	04:01:02 or 04:01 or 04:06	02:01:01	02:01:01	02:01 or 02:05	02:01 or 02:05
W5705	SAMN48396354	PCR-SBT	04:02:01 or 04:02:02	04:02:01 or 04:02:02	04:01:02 or 04:01 or 04:06	04:01:02 or 04:01 or 04:06	02:01:01	05:01 or 05:03	02:01 or 02:05	02:01 or 02:05
		NGSMHC	04:02:01 or 04:02:02	04:02:01 or 04:02:02	04:01:02 or 04:01 or 04:06	04:01:02 or 04:01 or 04:06	02:01:01	05:01 or 05:03	02:01 or 02:05	02:01 or 02:05
W5721	SAMN48396355	PCR-SBT	04:02:01 or 04:02:02	15:01	04:01:02 or 04:01 or 04:06	03:03	05:01 or 05:03	11:01	02:01 or 02:05	05:03
		NGSMHC	04:02:01 or 04:02:02	04:02:01 or 04:02:02	04:05	04:05	05:01 or 05:03	05:01 or 05:03	02:01 or 02:05	05:03

*SLA-2*’s low concordance due to paralog homology.

SLA, swine leukocyte antigen; PCR-SBT, polymerase chain reaction sequence-based typing; NGS, next-generation sequencing; MHC, major histocompatibility complex.

**Table 2:** Results of SLA typing of Babraham pig PacBio long-read sequencing data

SRA Run		SRR25949611	

Typing source		Data from Schwartz et al [31]	NGSMHC
*SLA-1*	Allele 1	14:02	14:02
	Allele 2	14:02	14:02
*SLA-2*	Allele 1	11:04 or 11:05^1)^	11:04 or 11:05
	Allele 2	11:04 or 11:05^1)^	11:04 or 11:05
*SLA-3*	Allele 1	04:03	04:02 or 04:03
	Allele 2	04:03	04:02 or 04:03
*SLA-DRA*	Allele 1	02:02:03	02:01:01 or 02:01:02 or 02:02:01 or 02:02:02 or 02:02:03
	Allele 2	02:02:03	02:01:01 or 02:01:02 or 02:02:01 or 02:02:02 or 02:02:03
*SLA-DRB1*	Allele 1	05:01	05:01 or 05:03
	Allele 2	05:01	05:01 or 05:03
*SLA-DQA*	Allele 1	01:03	01:03
	Allele 2	01:03	01:03
*SLA-DQB1*	Allele 1	08:01	08:01 or 08:02 or 08:04
	Allele 2	08:01	08:01 or 08:02 or 08:04

1) *SLA-2* typing results were based on BLASTN results from this study.

SLA, swine leukocyte antigen; NGS, next-generation sequencing; MHC, major histocompatibility complex.

## SUPPLEMENTARY MATERIAL

**Supplement 1**. Number of allele of target MHC allele references.

**Supplement 2**. Primer information for typing SLA genes.

**Supplement 3**. Sequencing quality metrics of Woori black pig short-read whole genome sequencing data.

**Supplement 4**. Flowchart for haplotype contig generation and subsequent MHC allele typing of NGSMHC pipeline.

**Supplement 5**. Sequencing and coverage characteristics of Woori black pig short read whole genome sequencing data.

**Supplement 6**. Sscrofa11.1 nucleotide BLAST results for *SLA-2* exon 2 and exon 3 allele sequences.

**Supplement 7**. Mapping region of reads with >99% sequence identity to *SLA-2*16:03* in W5161 whole genome sequencing data.

**Supplement 8**. BLASTN percent identity of exon 2 and 3 sequences from the target *SLA-2* allele against the TPI_Babraham_pig_v1 reference genome.

**Supplement 9**. Sequencing and coverage characteristics of Babraham pig long read whole genome sequencing data (SRR25949611).
