# Supplementary material for: NGSMHC: a simple bioinformatics tool for comprehensively typing major histocompatibility complex genes in non-human species using next-generation sequencing data
Source: Anim Biosci. 2025 Sep 30;39(2):250468. doi: 10.5713/ab.25.0468 (PMC12877382; doi:10.5713/ab.25.0468)
Supplement: Supplementary file 1 [file ab-25-0468-Supplementary-1.pdf]

Supplement 1. Number of allele of target MHC allele references

Species	SLA class	Gene	No. of allele	
			In IPD*	For analysis
pig	class I	*SLA-1*	100	85
		SLA-2	105	94
		SLA-3	47	39
		SLA-6	10	5
	class II	*SLA-DRA*	7	6
		SLA-DRB1	99	91
		SLA-DQA	27	23
		SLA-DQB1	55	45
		SLA-DMA	7	2

* Allele counts are based on the IPD database as of March 7, 2025.
