# Supplementary material for: NGSMHC: a simple bioinformatics tool for comprehensively typing major histocompatibility complex genes in non-human species using next-generation sequencing data
Source: Anim Biosci. 2025 Sep 30;39(2):250468. doi: 10.5713/ab.25.0468 (PMC12877382; doi:10.5713/ab.25.0468)
Supplement: Supplementary file 2 [file ab-25-0468-Supplementary-2.pdf]

Supplement 2. Primer information for typing SLA genes

Target loci	Primer usage	Sequence (5' to 3')	Amplicon size (bp)	Annealing (℃)	Reference
SLA-2 exon 2, 3	gPCR[1]	GCCTCGACACAGAATCTCCGATATATCCAAAGATG<br>CGGGTCACATGTGTCYTTGGAGG	1,679	65	Choi 2015
	Sequencing	AAKGCCSGGGTGACCCT<br>GACCCCCTTTTCCTCT	-	50	
	Additional Sequencing[2]	ATGCTGATTATCGCCCKCGTTGGWCGCG<br>TGCTATGCTGTGCGCCGARAGGAGGGT<br>GAGGGGAGATGGTGGAG<br>TTCCTGGGGATGGGGATG			
SLA-3 exon 2, 3	gPCR[1]	GGGGRCCCTGGCCCTGATT<br>CGGGTCACATGTGTCYTTGGAGG	1,655	65	Youk 2021
	Sequencing	AAKGCCSGGGTGACCCT<br>GACCCCCTTTTCCTCT	-	50	
	Additional Sequencing[2]	CTGTGAATGCTGCTGCGCCGAGAGGAGGGT<br>TGTGAATGCTATGGTKGGTCGYGGC			
*SLA-DRB*1 exon 2	gPCR[1]	GAATGCTGCGACTACCTGTGGATCATTGCTGTCCACGCAGMG<br>TCTACCAGGCATTCGCTTCATIIIIICYSCSGGCVGCSCA	364	65	Le 2011
	Sequencing	GAATGCTGCGACTAGACTGGATCATTGCTGTCCACGCATMG<br>TCTACCAGGCATTCGCTTCATIIIIICYSCSGGCVGCSCA	-	50	
SLA-DQB1 exon 2	gPCR[1]	GCGGCGGGTTTCAGGTGGATG<br>AACCCTCACTAAAGACCCACTCTCTCYGCGCGGWGTCTC	478	65	Le 2015
	Sequencing	AACCCTCACTAAAG	-	50	

[1] Primer for genomic PCR

[2] Additional sequencing primers for indel variants
