# Supplementary material for: NGSMHC: a simple bioinformatics tool for comprehensively typing major histocompatibility complex genes in non-human species using next-generation sequencing data
Source: Anim Biosci. 2025 Sep 30;39(2):250468. doi: 10.5713/ab.25.0468 (PMC12877382; doi:10.5713/ab.25.0468)
Supplement: Supplementary file 3 [file ab-25-0468-Supplementary-3.pdf]

Supplement 3. Sequencing quality metrics of Woori black pig short-read whole genome sequencing data

Sample	Yield (Bases)	Reads	% of >= Q30 Bases	Mean Quality Score
W4052	61,328,450,982	406,148,682	91.36	35.60
W4217	43,075,853,956	285,270,556	87.79	34.77
W4223	49,929,904,922	330,661,622	90.53	35.46
W5161	55,195,112,446	365,530,546	87.88	34.79
W5162	53,353,025,092	353,331,292	90.86	35.51
W4235	58,684,296,324	388,637,724	91.44	35.62
W4550	64,754,268,314	428,836,214	91.25	35.58
W4937	63,637,453,590	421,440,090	91.04	35.54
W5226	57,425,538,882	380,301,582	89.51	35.26
W5621	54,064,192,208	358,041,008	90.15	35.38
W5705	53,597,056,192	354,947,392	91.65	35.65
W5721	52,606,813,896	348,389,496	90.59	35.46
Average	55,637,663,900	368,461,350	90.34	35.39
