# Supplementary material for: NGSMHC: a simple bioinformatics tool for comprehensively typing major histocompatibility complex genes in non-human species using next-generation sequencing data
Source: Anim Biosci. 2025 Sep 30;39(2):250468. doi: 10.5713/ab.25.0468 (PMC12877382; doi:10.5713/ab.25.0468)
Supplement: Supplementary file 4 [file ab-25-0468-Supplementary-4.pdf]

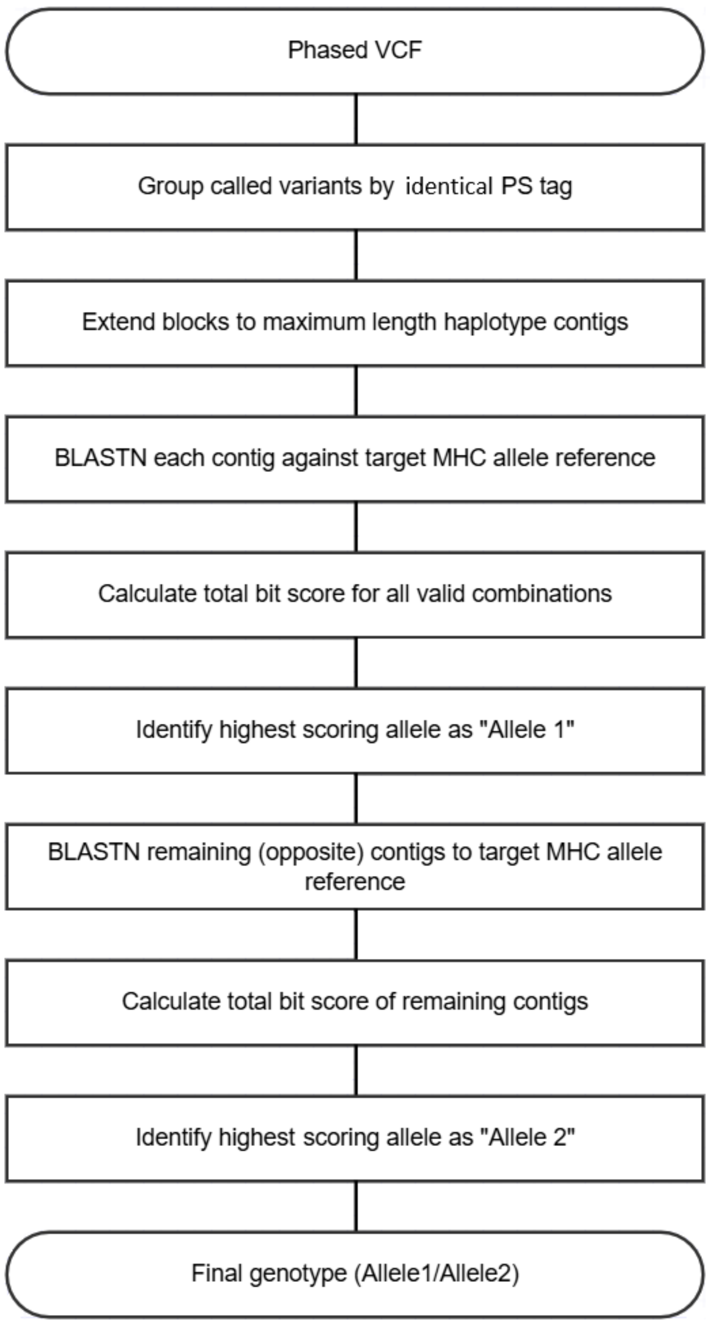

Supplement 4. Flowchart for haplotype contig generation and subsequent MHC allele typing of NGSMHC pipeline
