# Supplementary material for: NGSMHC: a simple bioinformatics tool for comprehensively typing major histocompatibility complex genes in non-human species using next-generation sequencing data
Source: Anim Biosci. 2025 Sep 30;39(2):250468. doi: 10.5713/ab.25.0468 (PMC12877382; doi:10.5713/ab.25.0468)
Supplement: Supplementary file 5 [file ab-25-0468-Supplementary-5.pdf]

Supplement 5. Sequencing and coverage characteristics of Woori black pig short read whole genome sequencing data

Category	Sample	Regions					
		SLA-2 e2	*SLA-2 e3*	*SLA-3 e2*	*SLA-3 e3*	*SLA-DRB1 e2*	*SLA-DQB1 e2*
No. of reads	W4052	22	137	94	75	59	82
	W4217	14	68	25	22	37	38
	W4223	18	170	73	78	46	60
	W5161	15	101	50	51	44	39
	W5162	18	93	38	33	28	47
	W4235	8	163	93	57	59	68
	W4550	17	108	79	42	56	99
	W4937	6	106	65	42	67	83
	W5226	15	128	63	75	49	56
	W5621	8	94	62	30	42	67
	W5705	7	64	52	41	28	42
	W5721	5	68	42	29	30	42
	Average	12.8	108.3	61.3	47.9	45.4	60.3
Breadth of coverage (%)	W4052	100.0	100.0	100.0	100.0	100.0	100.0
	W4217	100.0	100.0	100.0	100.0	100.0	100.0
	W4223	100.0	100.0	100.0	100.0	100.0	100.0
	W5161	100.0	100.0	100.0	100.0	100.0	100.0
	W5162	100.0	100.0	100.0	100.0	100.0	100.0
	W4235	100.0	100.0	100.0	100.0	100.0	100.0
	W4550	100.0	100.0	100.0	100.0	100.0	100.0
	W4937	86.7	100.0	100.0	100.0	100.0	100.0
	W5226	95.9	100.0	100.0	100.0	100.0	100.0
	W5621	100.0	100.0	100.0	100.0	100.0	100.0
	W5705	84.8	100.0	100.0	100.0	100.0	100.0
	W5721	100.0	100.0	100.0	100.0	100.0	100.0
	Average	97.3	100.0	100.0	100.0	100.0	100.0

Per-base read depth	W4052	8.9	50.4	36.8	22.5	19.6	29.2
	W4217	6.8	25.5	9.6	7.5	12.4	13.8
	W4223	6.8	67.0	28.0	28.3	18.0	19.3
	W5161	6.4	35.2	19.1	19.2	16.3	14.8
	W5162	8.5	39.9	14.4	11.2	10.4	17.1
	W4235	3.2	64.3	37.1	20.2	21.5	26.2
	W4550	7.4	39.9	32.1	13.3	18.5	32.3
	W4937	2.9	42.8	27.4	13.3	24.6	29.7
	W5226	6.6	51.1	26.7	25.1	21.0	18.7
	W5621	2.5	36.9	24.4	10.3	15.4	23.0
	W5705	2.1	26.5	21.5	13.4	9.7	16.7
	W5721	2.2	24.9	16.4	8.9	9.5	15.1
	Average	5.4	42.0	24.5	16.1	16.4	21.3
Mean base quality	W4052	35.0	35.9	35.1	35.6	35.3	35.4
	W4217	34.5	35.3	34.9	33.7	34.3	35.4
	W4223	34.4	35.9	34.7	35.5	35.0	35.3
	W5161	34.0	35.4	35.3	35.5	35.7	35.4
	W5162	33.7	35.6	35.0	35.4	35.2	35.6
	W4235	35.8	35.6	35.3	35.5	35.5	35.6
	W4550	34.4	36.0	35.4	35.9	35.4	34.4
	W4937	36.1	35.7	35.6	35.8	35.5	35.3
	W5226	34.4	35.5	34.9	35.7	35.6	34.6
	W5621	33.8	36.0	34.6	35.1	34.9	35.2
	W5705	36.3	35.8	35.7	35.7	36.1	35.6
	W5721	35.3	36.1	35.2	34.7	35.2	35.0
	Average	34.8	35.7	35.1	35.3	35.3	35.2
Mean map quality	W4052	47.6	50.4	48.4	40.5	68.7	60.0
	W4217	46.4	45.3	48.2	54.4	76.2	68.1
	W4223	48.4	44.2	44.4	41.9	70.1	70.6

W5161	49.1	51.2	46.6	46.5	63.5	73.2
W5162	43.1	45.9	59.3	48.4	64.9	71.3
W4235	32.1	49.0	50.8	45.8	64.0	60.0
W4550	30.4	44.3	49.9	39.6	60.0	70.7
W4937	32.5	49.4	53.2	38.2	58.0	61.4
W5226	48.5	48.3	57.5	43.0	59.4	68.9
W5621	33.2	45.6	52.5	44.4	59.1	61.4
W5705	13.3	47.4	44.4	44.6	70.7	60.0
W5721	34.4	46.6	51.5	42.4	76.4	74.9
Average	38.3	47.3	50.6	44.1	65.9	66.7
