# Supplementary material for: NGSMHC: a simple bioinformatics tool for comprehensively typing major histocompatibility complex genes in non-human species using next-generation sequencing data
Source: Anim Biosci. 2025 Sep 30;39(2):250468. doi: 10.5713/ab.25.0468 (PMC12877382; doi:10.5713/ab.25.0468)
Supplement: Supplementary file 6 [file ab-25-0468-Supplementary-6.pdf]

Supplement 6. Sscrofa11.1 nucleotide BLAST results for *SLA-2* exon 2 and exon 3 allele sequences

Region	query allele	subject genome	% identity	evalue	bit score	blastn in target
SLA-2 e2	*SLA-2*01:01*	NC_010449.5	100	2.25E-139	499	O
SLA-2 e2	*SLA-2*01:02*	NC_010449.5	100	2.25E-139	499	O
SLA-2 e2	*SLA-2*01:03*	NC_010449.5	98.148	4.91E-131	472	O
SLA-2 e2	*SLA-2*02:01*	NC_010449.5	90.741	1.11E-97	361	X
SLA-2 e2	*SLA-2*02:02*	NC_010449.5	91.852	1.10E-102	377	X
SLA-2 e2	*SLA-2*02:03*	NC_010449.5	87.037	5.27E-81	305	X
SLA-2 e2	*SLA-2*02:04*	NC_010449.5	90.741	1.11E-97	361	X
SLA-2 e2	*SLA-2*02:05*	NC_010449.5	90.741	1.11E-97	361	X
SLA-2 e2	*SLA-2*03:01*	NC_010449.5	93.358	2.35E-109	399	X
SLA-2 e2	*SLA-2*03:02*	NC_010449.5	93.727	5.05E-111	405	O
SLA-2 e2	*SLA-2*03:03*	NC_010449.5	93.727	5.05E-111	405	O
SLA-2 e2	*SLA-2*04:01*	NC_010449.5	93.015	1.09E-107	394	X
SLA-2 e2	*SLA-2*04:02:01_or_SLA-2*04:02:02*	NC_010449.5	93.015	1.09E-107	394	X
SLA-2 e2	*SLA-2*04:03*	NC_010449.5	95.572	2.32E-119	433	X
SLA-2 e2	*SLA-2*04:04*	NC_010449.5	95.572	2.32E-119	433	X
SLA-2 e2	*SLA-2*04:05*	NC_010449.5	93.015	1.09E-107	394	X
SLA-2 e2	*SLA-2*04:06*	NC_010449.5	91.912	1.10E-102	377	X
SLA-2 e2	*SLA-2*04:07*	NC_010449.5	93.015	1.09E-107	394	X
SLA-2 e2	*SLA-2*04:08*	NC_010449.5	91.544	5.12E-101	372	X
SLA-2 e2	*SLA-2*04:09*	NC_010449.5	95.941	4.98E-121	438	X
SLA-2 e2	*SLA-2*05:01*	NC_010449.5	93.727	5.05E-111	405	X
SLA-2 e2	*SLA-2*05:02*	NC_010449.5	92.279	2.37E-104	383	X
SLA-2 e2	*SLA-2*05:03*	NC_010449.5	92.989	1.09E-107	394	X
SLA-2 e2	*SLA-2*05:04*	NC_010449.5	93.015	1.09E-107	394	O
SLA-2 e2	*SLA-2*05:05*	NC_010449.5	93.015	1.09E-107	394	O
SLA-2 e2	*SLA-2*05:06*	NC_010449.5	93.727	5.05E-111	405	X
SLA-2 e2	*SLA-2*05:07*	NC_010449.5	92.647	5.09E-106	388	X
SLA-2 e2	*SLA-2*05:08*	NC_010449.5	92.989	1.09E-107	394	O

SLA-2 e2	*SLA-2*05:09*	NC_010449.5	93.015	1.09E-107	394	O
SLA-2 e2	*SLA-2*06:01*	NC_010449.5	89.299	5.20E-91	339	X
SLA-2 e2	*SLA-2*06:02:01_or_SLA-2*06:02:02*	NC_010449.5	91.144	2.38E-99	366	X
SLA-2 e2	*SLA-2*06:03*	NC_010449.5	90.741	1.11E-97	361	X
SLA-2 e2	*SLA-2*06:04*	NC_010449.5	92.222	2.37E-104	383	X
SLA-2 e2	*SLA-2*06:05*	NC_010449.5	91.481	5.12E-101	372	X
SLA-2 e2	*SLA-2*06:06*	NC_010449.5	91.111	2.38E-99	366	X
SLA-2 e2	*SLA-2*06:07*	NC_010449.5	89.299	5.20E-91	339	X
SLA-2 e2	*SLA-2*06:08*	NC_010449.5	90.406	5.16E-96	355	X
SLA-2 e2	*SLA-2*06:09*	NC_010449.5	90.37	5.16E-96	355	X
SLA-2 e2	*SLA-2*06:10*	NC_010449.5	90.406	5.16E-96	355	X
SLA-2 e2	*SLA-2*06:11*	NC_010449.5	89.63	1.12E-92	344	X
SLA-2 e2	*SLA-2*06:12*	NC_010449.5	89.63	1.12E-92	344	X
SLA-2 e2	*SLA-2*06:13*	NC_010449.5	90.37	5.16E-96	355	X
SLA-2 e2	*SLA-2*06:14*	NC_010449.5	91.481	5.12E-101	372	X
SLA-2 e2	*SLA-2*06:15*	NC_010449.5	90.741	1.11E-97	361	X
SLA-2 e2	*SLA-2*06:16*	NC_010449.5	89.299	5.20E-91	339	X
SLA-2 e2	*SLA-2*07:01*	NC_010449.5	97.778	2.28E-129	466	O
SLA-2 e2	*SLA-2*07:02*	NC_010449.5	97.778	2.28E-129	466	O
SLA-2 e2	*SLA-2*07:03*	NC_010449.5	98.889	2.27E-134	483	O
SLA-2 e2	*SLA-2*07:04*	NC_010449.5	98.889	2.27E-134	483	O
SLA-2 e2	*SLA-2*07:05*	NC_010449.5	93.704	5.05E-111	405	O
SLA-2 e2	*SLA-2*07:06*	NC_010449.5	97.778	2.28E-129	466	O
SLA-2 e2	*SLA-2*08:01*	NC_010449.5	92.593	5.09E-106	388	X
SLA-2 e2	*SLA-2*08:02_or_SLA-2*08:03*	NC_010449.5	92.593	5.09E-106	388	X
SLA-2 e2	*SLA-2*08:04*	NC_010449.5	93.333	2.35E-109	399	X
SLA-2 e2	*SLA-2*08:05*	NC_010449.5	91.852	1.10E-102	377	X
SLA-2 e2	*SLA-2*08:06*	NC_010449.5	92.593	5.09E-106	388	X
SLA-2 e2	*SLA-2*08:07*	NC_010449.5	92.593	5.09E-106	388	X
SLA-2 e2	*SLA-2*09:01*	NC_010449.5	95.556	2.32E-119	433	X

SLA-2 e2	*SLA-2*09:02*	NC_010449.5	95.556	2.32E-119	433	X
SLA-2 e2	*SLA-2*09:03*	NC_010449.5	95.203	1.08E-117	427	X
SLA-2 e2	*SLA-2*09:04*	NC_010449.5	96.667	2.30E-124	449	X
SLA-2 e2	*SLA-2*09:06*	NC_010449.5	94.444	2.33E-114	416	X
SLA-2 e2	*SLA-2*10:01*	NC_010449.5	92.593	5.09E-106	388	X
SLA-2 e2	*SLA-2*10:02*	NC_010449.5	90.741	1.11E-97	361	X
SLA-2 e2	*SLA-2*10:03*	NC_010449.5	91.481	5.12E-101	372	X
SLA-2 e2	*SLA-2*10:04*	NC_010449.5	91.111	2.38E-99	366	X
SLA-2 e2	*SLA-2*10:05*	NC_010449.5	90.741	1.11E-97	361	X
SLA-2 e2	*SLA-2*10:06*	NC_010449.5	90.741	1.11E-97	361	X
SLA-2 e2	*SLA-2*10:07*	NC_010449.5	91.111	2.38E-99	366	X
SLA-2 e2	*SLA-2*10:08*	NC_010449.5	92.222	2.37E-104	383	X
SLA-2 e2	*SLA-2*10:09*	NC_010449.5	90.741	1.11E-97	361	X
SLA-2 e2	*SLA-2*11:01:01*	NC_010449.5	90.476	5.16E-96	355	X
SLA-2 e2	*SLA-2*11:01:02*	NC_010449.5	90.476	5.16E-96	355	X
SLA-2 e2	*SLA-2*11:02*	NC_010449.5	91.513	5.12E-101	372	X
SLA-2 e2	*SLA-2*11:03*	NC_010449.5	90.476	5.16E-96	355	X
SLA-2 e2	*SLA-2*11:04_or_SLA-2*11:05*	NC_010449.5	93.04	1.09E-107	394	O
SLA-2 e2	*SLA-2*12:01*	NC_010449.5	97.778	2.28E-129	466	O
SLA-2 e2	*SLA-2*12:02*	NC_010449.5	97.778	2.28E-129	466	O
SLA-2 e2	*SLA-2*13:01*	NC_010449.5	96.296	1.07E-122	444	O
SLA-2 e2	*SLA-2*13:02*	NC_010449.5	96.296	1.07E-122	444	O
SLA-2 e2	*SLA-2*13:03*	NC_010449.5	95.926	4.98E-121	438	O
SLA-2 e2	*SLA-2*14:01*	NC_010449.5	90.406	5.16E-96	355	X
SLA-2 e2	*SLA-2*15:01*	NC_010449.5	95.185	1.08E-117	427	X
SLA-2 e2	*SLA-2*16:01*	NC_010449.5	91.882	1.10E-102	377	X
SLA-2 e2	*SLA-2*16:02*	NC_010449.5	90.809	1.11E-97	361	X
SLA-2 e2	*SLA-2*16:03*	NC_010449.5	90.809	1.11E-97	361	X
SLA-2 e2	*SLA-2*17:01*	NC_010449.5	90.775	1.11E-97	361	X
SLA-2 e2	*SLA-2*18:01*	NC_010449.5	93.727	5.05E-111	405	O

SLA-2 e2	*SLA-2*18:02*	NC_010449.5	91.144	2.38E-99	366	X
SLA-2 e2	*SLA-2*18:03*	NC_010449.5	89.011	2.42E-89	333	X
SLA-2 e2	*SLA-2*19:01*	NC_010449.5	95.185	1.08E-117	427	X
SLA-2 e2	*SLA-2*20:01*	NC_010449.5	91.882	1.10E-102	377	X
SLA-2 e2	*SLA-2*21:01*	NC_010449.5	96.296	1.07E-122	444	X
SLA-2 e2	*SLA-2*GZ14*	NC_010449.5	91.481	5.12E-101	372	X
SLA-2 e3	*SLA-2*01:01*	NC_010449.5	96.751	1.09E-127	460	O
SLA-2 e3	*SLA-2*01:02*	NC_010449.5	100	1.07E-142	510	O
SLA-2 e3	*SLA-2*01:03*	NC_010449.5	98.551	5.00E-136	488	O
SLA-2 e3	*SLA-2*02:01*	NC_010449.5	92.029	5.21E-106	388	O
SLA-2 e3	*SLA-2*02:02*	NC_010449.5	91.667	2.43E-104	383	O
SLA-2 e3	*SLA-2*02:03*	NC_010449.5	92.029	5.21E-106	388	O
SLA-2 e3	*SLA-2*02:04*	NC_010449.5	94.565	1.10E-117	427	X
SLA-2 e3	*SLA-2*02:05*	NC_010449.5	97.112	2.34E-129	466	O
SLA-2 e3	*SLA-2*03:01*	NC_010449.5	95.29	5.10E-121	438	X
SLA-2 e3	*SLA-2*03:02*	NC_010449.5	95.29	5.10E-121	438	X
SLA-2 e3	*SLA-2*03:03*	NC_010449.5	95.652	1.10E-122	444	O
SLA-2 e3	*SLA-2*04:01*	NC_010449.5	97.112	2.34E-129	466	O
SLA-2 e3	*SLA-2*04:02:01_or_SLA-2*04:02:02*	NC_010449.5	97.101	2.34E-129	466	O
SLA-2 e3	*SLA-2*04:03*	NC_010449.5	95.307	5.10E-121	438	O
SLA-2 e3	*SLA-2*04:04*	NC_010449.5	95.307	5.10E-121	438	O
SLA-2 e3	*SLA-2*04:05*	NC_010449.5	97.101	2.34E-129	466	O
SLA-2 e3	*SLA-2*04:06*	NC_010449.5	96.377	5.07E-126	455	O
SLA-2 e3	*SLA-2*04:07*	NC_010449.5	92.029	5.21E-106	388	O
SLA-2 e3	*SLA-2*04:08*	NC_010449.5	92.754	2.41E-109	399	O
SLA-2 e3	*SLA-2*04:09*	NC_010449.5	97.464	5.03E-131	472	O
SLA-2 e3	*SLA-2*05:01*	NC_010449.5	93.525	1.11E-112	411	O
SLA-2 e3	*SLA-2*05:02*	NC_010449.5	93.478	1.11E-112	411	O
SLA-2 e3	*SLA-2*05:03*	NC_010449.5	92.754	2.41E-109	399	O
SLA-2 e3	*SLA-2*05:04*	NC_010449.5	92.754	2.41E-109	399	O

SLA-2 e3	*SLA-2*05:05*	NC_010449.5	94.928	2.37E-119	433	O
SLA-2 e3	*SLA-2*05:06*	NC_010449.5	92.806	2.41E-109	399	O
SLA-2 e3	*SLA-2*05:07*	NC_010449.5	95.307	5.10E-121	438	O
SLA-2 e3	*SLA-2*05:08*	NC_010449.5	95.307	5.10E-121	438	O
SLA-2 e3	*SLA-2*05:09*	NC_010449.5	94.928	2.37E-119	433	O
SLA-2 e3	*SLA-2*06:01*	NC_010449.5	94.946	2.37E-119	433	O
SLA-2 e3	*SLA-2*06:02:01_or_SLA-2*06:02:02*	NC_010449.5	92.754	2.41E-109	399	X
SLA-2 e3	*SLA-2*06:03*	NC_010449.5	94.245	5.14E-116	422	O
SLA-2 e3	*SLA-2*06:04*	NC_010449.5	94.928	2.37E-119	433	X
SLA-2 e3	*SLA-2*06:05*	NC_010449.5	93.841	2.39E-114	416	X
SLA-2 e3	*SLA-2*06:06*	NC_010449.5	92.78	2.41E-109	399	X
SLA-2 e3	*SLA-2*06:07*	NC_010449.5	93.885	2.39E-114	416	O
SLA-2 e3	*SLA-2*06:08*	NC_010449.5	94.203	5.14E-116	422	X
SLA-2 e3	*SLA-2*06:09*	NC_010449.5	95.668	1.10E-122	444	X
SLA-2 e3	*SLA-2*06:10*	NC_010449.5	92.754	2.41E-109	399	X
SLA-2 e3	*SLA-2*06:11*	NC_010449.5	93.478	1.11E-112	411	X
SLA-2 e3	*SLA-2*06:12*	NC_010449.5	92.754	2.41E-109	399	X
SLA-2 e3	*SLA-2*06:13*	NC_010449.5	92.754	2.41E-109	399	X
SLA-2 e3	*SLA-2*06:14*	NC_010449.5	93.478	1.11E-112	411	X
SLA-2 e3	*SLA-2*06:15*	NC_010449.5	95.29	5.10E-121	438	O
SLA-2 e3	*SLA-2*06:16*	NC_010449.5	92.029	5.21E-106	388	X
SLA-2 e3	*SLA-2*07:01*	NC_010449.5	92.029	5.21E-106	388	O
SLA-2 e3	*SLA-2*07:02*	NC_010449.5	90.58	2.44E-99	366	O
SLA-2 e3	*SLA-2*07:03*	NC_010449.5	92.391	1.12E-107	394	O
SLA-2 e3	*SLA-2*07:04*	NC_010449.5	95.652	1.10E-122	444	O
SLA-2 e3	*SLA-2*07:05*	NC_010449.5	94.565	1.10E-117	427	O
SLA-2 e3	*SLA-2*07:06*	NC_010449.5	90.942	5.25E-101	372	O
SLA-2 e3	*SLA-2*08:01*	NC_010449.5	99.275	2.31E-139	499	O
SLA-2 e3	*SLA-2*08:02_or_SLA-2*08:03*	NC_010449.5	95.307	5.10E-121	438	X
SLA-2 e3	*SLA-2*08:04*	NC_010449.5	94.224	5.14E-116	422	X

SLA-2 e3	*SLA-2*08:05*	NC_010449.5	93.863	2.39E-114	416	O
SLA-2 e3	*SLA-2*08:06*	NC_010449.5	95.307	5.10E-121	438	O
SLA-2 e3	*SLA-2*08:07*	NC_010449.5	94.224	5.14E-116	422	X
SLA-2 e3	*SLA-2*09:01*	NC_010449.5	94.565	1.10E-117	427	X
SLA-2 e3	*SLA-2*09:02*	NC_010449.5	95.29	5.10E-121	438	X
SLA-2 e3	*SLA-2*09:03*	NC_010449.5	94.203	5.14E-116	422	X
SLA-2 e3	*SLA-2*09:04*	NC_010449.5	94.203	5.14E-116	422	X
SLA-2 e3	*SLA-2*09:06*	NC_010449.5	94.203	5.14E-116	422	X
SLA-2 e3	*SLA-2*10:01*	NC_010449.5	95.652	1.10E-122	444	X
SLA-2 e3	*SLA-2*10:02*	NC_010449.5	94.928	2.37E-119	433	O
SLA-2 e3	*SLA-2*10:03*	NC_010449.5	96.739	1.09E-127	460	X
SLA-2 e3	*SLA-2*10:04*	NC_010449.5	96.739	1.09E-127	460	X
SLA-2 e3	*SLA-2*10:05*	NC_010449.5	94.565	1.10E-117	427	X
SLA-2 e3	*SLA-2*10:06*	NC_010449.5	95.652	1.10E-122	444	O
SLA-2 e3	*SLA-2*10:07*	NC_010449.5	92.754	2.41E-109	399	X
SLA-2 e3	*SLA-2*10:08*	NC_010449.5	95.652	1.10E-122	444	X
SLA-2 e3	*SLA-2*10:09*	NC_010449.5	94.928	2.37E-119	433	O
SLA-2 e3	*SLA-2*11:01:01*	NC_010449.5	93.841	2.39E-114	416	X
SLA-2 e3	*SLA-2*11:01:02*	NC_010449.5	94.565	1.10E-117	427	X
SLA-2 e3	*SLA-2*11:02*	NC_010449.5	95.29	5.10E-121	438	O
SLA-2 e3	*SLA-2*11:03*	NC_010449.5	94.928	2.37E-119	433	O
SLA-2 e3	*SLA-2*11:04_or_SLA-2*11:05*	NC_010449.5	93.116	5.18E-111	405	X
SLA-2 e3	*SLA-2*12:01*	NC_010449.5	93.841	2.39E-114	416	X
SLA-2 e3	*SLA-2*12:02*	NC_010449.5	93.478	1.11E-112	411	X
SLA-2 e3	*SLA-2*13:01*	NC_010449.5	96.739	1.09E-127	460	O
SLA-2 e3	*SLA-2*13:02*	NC_010449.5	95.652	1.10E-122	444	O
SLA-2 e3	*SLA-2*13:03*	NC_010449.5	96.739	1.09E-127	460	O
SLA-2 e3	*SLA-2*14:01*	NC_010449.5	93.841	2.39E-114	416	X
SLA-2 e3	*SLA-2*15:01*	NC_010449.5	95.29	5.10E-121	438	O
SLA-2 e3	*SLA-2*16:01*	NC_010449.5	94.585	1.10E-117	427	O

SLA-2 e3	*SLA-2*16:02*	NC_010449.5	94.224	5.14E-116	422	X
SLA-2 e3	*SLA-2*16:03*	NC_010449.5	94.565	1.10E-117	427	O
SLA-2 e3	*SLA-2*17:01*	NC_010449.5	90.942	5.25E-101	372	X
SLA-2 e3	*SLA-2*18:01*	NC_010449.5	96.014	2.36E-124	449	X
SLA-2 e3	*SLA-2*18:02*	NC_010449.5	94.203	5.14E-116	422	X
SLA-2 e3	*SLA-2*18:03*	NC_010449.5	93.116	5.18E-111	405	O
SLA-2 e3	*SLA-2*19:01*	NC_010449.5	94.565	1.10E-117	427	X
SLA-2 e3	*SLA-2*20:01*	NC_010449.5	96.029	2.36E-124	449	O
SLA-2 e3	*SLA-2*21:01*	NC_010449.5	96.377	5.07E-126	455	O
SLA-2 e3	*SLA-2*GZ14*	NC_010449.5	93.478	1.11E-112	411	X
