# Supplementary material for: NGSMHC: a simple bioinformatics tool for comprehensively typing major histocompatibility complex genes in non-human species using next-generation sequencing data
Source: Anim Biosci. 2025 Sep 30;39(2):250468. doi: 10.5713/ab.25.0468 (PMC12877382; doi:10.5713/ab.25.0468)
Supplement: Supplementary file 7 [file ab-25-0468-Supplementary-7.pdf]

Supplement 7. Mapping region of reads with >99% sequence identity to *SLA-2*16:03* in W5161 whole genome sequencing data

Read ID	Mapping region		Corresponding region
	Chr.	Start pos.	
A00939:277:HCTT5DSX7:1:2330:3974:18991	7	22824772	*SLA-1*
A00939:277:HCTT5DSX7:1:2537:3902:6339	7	22889192	*SLA-1*
A00939:277:HCTT5DSX7:1:2437:7003:16501	7	22889241	*SLA-1*
A00939:277:HCTT5DSX7:1:1322:18050:15749	7	22824850	*SLA-1*
A00939:277:HCTT5DSX7:1:2411:14073:2503	7	22956423	*SLA-2*
A00939:277:HCTT5DSX7:1:2331:7916:3740	7	22824873	*SLA-1*
A00939:277:HCTT5DSX7:1:2167:8703:3912	7	22824872	*SLA-1*
A00939:277:HCTT5DSX7:1:2537:19849:11757	7	22889449	*SLA-1*
A00939:277:HCTT5DSX7:1:2245:9851:3176	7	22889452	*SLA-1*
A00939:277:HCTT5DSX7:1:2161:25446:24956	7	22889465	*SLA-1*
A00939:277:HCTT5DSX7:1:2245:9815:3145	7	22889452	*SLA-1*
A00939:277:HCTT5DSX7:1:2103:29243:22670	7	22889477	*SLA-1*
A00939:277:HCTT5DSX7:1:1156:21206:20901	7	22889481	*SLA-1*
A00939:277:HCTT5DSX7:1:2205:27905:24612	7	22938928	*SLA-3*
A00939:277:HCTT5DSX7:1:2447:4625:15139	7	22825059	*SLA-1*
A00939:277:HCTT5DSX7:1:2518:28800:11600	7	22825061	*SLA-1*
A00939:277:HCTT5DSX7:1:2351:21739:15781	7	22889544	*SLA-1*
A00939:277:HCTT5DSX7:1:1410:8883:33630	7	22956664	*SLA-2*
A00939:277:HCTT5DSX7:1:2205:9218:6746	7	22956656	*SLA-2*
A00939:277:HCTT5DSX7:1:2558:27706:24079	7	22868711	*SLA-1*
A00939:277:HCTT5DSX7:1:1305:12590:12680	7	22868694	*SLA-1*
A00939:277:HCTT5DSX7:1:2113:18502:21010	7	22825056	*SLA-1*
A00939:277:HCTT5DSX7:1:2328:23457:35102	7	22825050	*SLA-1*
A00939:277:HCTT5DSX7:1:2567:8323:1407	7	22889550	*SLA-1*
A00939:277:HCTT5DSX7:1:1108:25617:22060	7	22868717	*SLA-1*
A00939:277:HCTT5DSX7:1:1270:17336:20149	7	23637671	*SLA-7*

A00939:277:HCTT5DSX7:1:1265:10113:32753	7	23637679	*SLA-7*
