# Supplementary material for: NGSMHC: a simple bioinformatics tool for comprehensively typing major histocompatibility complex genes in non-human species using next-generation sequencing data
Source: Anim Biosci. 2025 Sep 30;39(2):250468. doi: 10.5713/ab.25.0468 (PMC12877382; doi:10.5713/ab.25.0468)
Supplement: Supplementary file 8 [file ab-25-0468-Supplementary-8.pdf]

Supplement 8. BLASTN percent identity of exon 2 and 3 sequences from the target SLA-2 allele against the TPI_Babraham_pig_v1 reference genome

Query	Subject	% identity
*SLA-2*11:02_e2*	CM062304.1	93.4
*SLA-2*11:02_e3*	CM062304.1	94.6
*SLA-2*11:04_or_SLA-2*11:05_e2*	CM062304.1	100.0
*SLA-2*11:04_or_SLA-2*11:05_e3*	CM062304.1	100.0
