# Supplementary material for: NGSMHC: a simple bioinformatics tool for comprehensively typing major histocompatibility complex genes in non-human species using next-generation sequencing data
Source: Anim Biosci. 2025 Sep 30;39(2):250468. doi: 10.5713/ab.25.0468 (PMC12877382; doi:10.5713/ab.25.0468)
Supplement: Supplementary file 9 [file ab-25-0468-Supplementary-9.pdf]

Supplement 9. Sequencing and coverage characteristics of Babraham pig long read whole genome sequencing data (SRR25949611)

Category	Regions									
	SLA-1 e2	*SLA-1 e3*	*SLA-2 e2*	*SLA-2 e3*	*SLA-3 e2*	*SLA-3 e3*	*SLA-DRA e2*	*SLA-DRB1 e2*	*SLA-DQA e2*	*SLA-DQB1 e2*
No. of reads	16	17	17	18	23	15	18	12	19	19
Breadth of coverage (%)	100.0	100.0	100.0	100.0	100.0	100.0	100.0	100.0	100.0	100.0
Per-base read depth	14.9	14.9	15.2	17.2	21.9	14.3	17.3	10.8	18.2	17.2
Mean map quality	58.0	58.1	60.0	60.0	58.9	58.9	60.0	60.0	60.0	58.1
